# LINC00665 knockdown confers sensitivity in irradiated non-small cell lung cancer cells through the miR-582-5p/UCHL3/AhR axis

**DOI:** 10.1186/s12967-022-03516-2

**Published:** 2022-08-02

**Authors:** Li-Ming Xu, Ya-Jing Yuan, Hao Yu, Shuai Wang, Ping Wang

**Affiliations:** 1 Department of Radiotherapy, Key Laboratory of Cancer Prevention and Therapy, Tianjin Medical University Cancer Institute & Hospital, National Clinical Research Center for Cancer, Tianjin’s Clinical Research Center for Cancer, West Huanhu Road, Tiyuanbei, Hexi District, Tianjin, 300060 People’s Republic of China; 2 Department of Anesthesia, Key Laboratory of Cancer Prevention and Therapy, Tianjin Medical University Cancer Institute & Hospital, National Clinical Research Center for Cancer, Tianjin’s Clinical Research Center for Cancer, Tianjin, 300060 People’s Republic of China; 3 Department of Hepatobiliary Oncology, Key Laboratory of Cancer Prevention and Therapy, Tianjin Medical University Cancer Institute & Hospital, National Clinical Research Center for Cancer, Tianjin’s Clinical Research Center for Cancer, Tianjin, 300060 People’s Republic of China

**Keywords:** LINC00665, Non-small cell lung cancer, microRNA-582-5p, Ubiquitin C-terminal hydrolase L3, Aryl hydrocarbon receptor, Deubiquitination, Radiosensitivity, Immune escape

## Abstract

**Background:**

The resistance to radiotherapy remains a major obstacle that limits the efficacy of radiotherapy in non-small cell lung cancer (NSCLC). This study aims to illustrate the molecular mechanism underlying the role of LINC00665 in the radiosensitivity of NSCLC, which involves ubiquitin C-terminal hydrolase L3 (UCHL3).

**Methods and results:**

The expression of UCHL3 was determined in clinical tissue samples collected from NSCLC patients and NSCLC cell lines. We found that UCHL3 overexpression occurred in both NSCLC tissues and cells, associated with poor prognosis in NSCLC patients. Mechanistically, UCHL3 stabilized aryl hydrocarbon receptor (AhR) protein through deubiquitination, thereby promoting PD-L1 expression. UCHL3 reduced the radiosensitivity of NSCLC cells by stabilizing AhR protein. Upstream microRNAs (miRNAs) and lncRNAs of UCHL3 were predicted by microarray profiling and validated by functional experiments. LINC00665 functioned as a sponge of miR-582-5p and thus up-regulated the expression of the miR-582-5p target UCHL3. Gain- and loss- of function assays were performed to assess the effects of LINC00665, UCHL3 and miR-582-5p on the in vitro cell malignant behaviors and immune escape as well as on the in vivo tumor growth. Silencing LINC00665 or overexpressing miR-582-5p enhanced the sensitivity of NSCLC cells to radiotherapy. LINC00665 augmented the immune escape of NSCLC cells in vitro and in vivo through stabilizing AhR protein via the miR-582-5p/UCHL3 axis.

**Conclusions:**

Overall, LINC00665 reduced the radiosensitivity of NSCLC cells via stabilization of AhR through the miR-582-5p/UCHL3 axis.

**Supplementary Information:**

The online version contains supplementary material available at 10.1186/s12967-022-03516-2.

## Background

Lung cancer remains the leading cause of cancer-related mortality worldwide, and non-small cell lung cancer (NSCLC) represents the most common type, accounting for more than two-thirds of lung cancer cases [1, 2]. In the multidisciplinary management of NSCLC, radiotherapy is the most commonly used nonsurgical therapeutic method [3]. Apart from DNA damage, radiation-induced antitumor processes includes the modulation of the tumor microenvironment, wherein the host immune response to the tumor site was triggered through recruitment of antigen-presenting cells and activation of tumor antigen-specific T-cell responses [4, 5]. Nonetheless, the durable of the antitumor immune response to radiotherapy is impeded by the frequent immune escape, which generally develops with tumor relapse [6, 7]. Herein, investigations of novel molecules and regulatory mechanisms underlying the immune escape are required for boosting the efficacy of radiotherapy in NSCLC.

Protein ubiquitination, as a highly regulated posttranscriptional modification, can be reversed by reactions catalyzed by deubiquitinating enzymes (DUBs), which remove ubiquitin from protein substrates and, generally, prevent degradation of proteins [8]. DUBs identified to date are classified into five subfamilies, one of which is the ubiquitin C-terminal hydrolase (UCH) family [9, 10]. Intriguingly, UCHL3, as a member of UCH family, has recently been revealed to be highly expressed in NSCLC, maintaining the cancer stem-like properties through stabilizing the aryl hydrocarbon receptor (AhR) for γ-H2ax [11]. AhR is a transcription factor of the basic helix-loop/PER-ARNT-SIM family, and members of this family require ligand activation [12]. Interestingly, AhR has been highlighted to enhance the immune escape of lung epithelial cells in radiotherapy by contributing to the tobacco-induced upregulation of PD-L1 [13].

To explore the up-restream mechanism of the potential UCHL3/AhR/PD-L1 axis, we conducted bioinformatics analysis and predicted that LINC00665 may sponge microRNA (miR)-582-5p to mediate UCHL3. Accumulating evidence has demonstrated the critical roles of long non-coding RNAs (lncRNAs), namely transcripts larger than 200 nucleotides with little protein-coding potential, in biological processes of cancer cells in a wide spectrum of cancers [14]. Of note, the involvement of oncogenic LINC00665 in the progression of lung cancers has previously been documented [15, 16]; the suppression of LINC00665 restricts malignant phenotypes of NSCLC cells via modulating a miRNA-dependent signaling [17]. Moreover, miR-582-5p has recently been established to exert tumor-inhibiting activities in NSCLC [18].

Therefore, we hypothesized in this work that the LINC00665/miR-582-5p/UCHL3 cascade may affect the radiotherapy sensitivity and immune escape in NSCLC, which might involve AhR protein stability and PD-L1 expression.

## Materials and methods

### Ethics statement

The study protocol was approved by the Ethics Committee of Tianjin Medical University Cancer Institute & Hospital and conducted strictly in accordance with the Declaration of Helsinki. Signed informed consents were provided by all participants. Animal experiments were in line with the Guide for the Care and Use of Laboratory Animals published by US National Institutes of Health.

### Microarray-based gene expression profiling

Datasets of GSE48414 (20 normal lung tissue samples and 154 NSCLC tissue samples), GSE15008 (188 normal lung tissue samples and 187 NSCLC tissue samples) and GSE20549 (21 cases of the radiotherapy-resistant H1299 lung cancer cell line as well as 21 cases of the radiotherapy-sensitive H460 lung cancer cell line) were retrieved from the GEO database. TCGA-based RNA sequencing data for lung adenocarcinoma (LUAD) (526 LUAD samples and 59 normal lung tissue samples) and lung squamous cell carcinoma (LUSC) (501 LUSC samples and 49 normal lung tissue samples) were obtained from the UCSC Xena database. Limma package in R language was utilized to screen differentially expressed genes with |logFC|> 1 and *p* < 0.05 as the threshold. Further, the GEPIA database was utilized for analyses of the expression of candidate genes in NSCLC. TargetScan and ENCORI databases were employed to analyze miRNA-mRNA interactions. LncRNA targeted by the candidate miRNA was predicted based on the LncBase database.

### Patient enrollment

A total of 45 NSCLC patients (31 males and 14 females, aged 39—78 years with a mean age of 59.4 years) who underwent surgical treatment at Tianjin Medical University Cancer Institute & Hospital from January 2018 to January 2019 were enrolled in the present study. NSCLC tumor tissues and adjacent normal tissues were harvested. None of the patients received preoperative chemotherapy or radiotherapy. The histopathological characteristics of NSCLC tumors were assessed according to the 8th edition of the American Joint Committee on Cancer (AJCC) staging system. A part of the tissues was quickly frozen in liquid nitrogen, while the other part was fixed in 10% neutral buffered formalin and stored at − 80 °C.

### RNA extraction and gene expression measurement

After total RNA extraction from NSCLC tissues and cells utilizing TRIzol reagent (16096020, Thermo Fisher Scientific, New York, NY), the total RNA of mRNA and lncRNA was reversely transcribed into cDNA using a reverse transcription kit (RR047A, Takara Bio, Shiga, Japan). The production of cDNA of miRNA was conducted by a PolyA tailing detection kit (B532451, Sangon Biotech, Shanghai, China). SYBR® Premix Ex TaqTM II kit (DRR081, Takara) was used for loading, and the samples were subjected to qRT-PCR in a fluorescent qRT-PCR system (ABI 7500, ABI, Foster City, CA). The 2^−ΔΔCt^ method was used for the quantification of the relative expression of target genes, with U6 and GAPDH serving as housekeeping genes (Additional file 1: Table S1).

### Immunohistochemistry

Paraffin-embedded Sections (4 μm thick) of tissues were subjected to antigen retrieval in EDTA buffer, followed by incubation with 0.3% H_2_O_2_. After blocking with 5% BSA, samples were immunoblotted at 4 °C overnight with primary antibodies to UCHL3 (1:100, ab241490, Abcam, Cambridge, UK), AhR (1: 200, MA1-513, Thermo Fisher Scientific) and ki67 (1:100, ab15580, Abcam). Subsequently, the sections were incubated with the biotinylated secondary antibody goat anti-rabbit IgG (1:1000, ab6721, Abcam) or goat anti-mouse IgG (1: 500, 31430, Thermo Fisher Scientific), followed by another incubation with HRP-labeled streptavidin (Innova Biosciences, Cambridge, UK). After DAB development and hematoxylin counterstaining, the sections were observed using a microscope (Leica-DM2500, Leica, Bensheim, Germany), and images were photographed by ImagePro Plus 7.1 software.

### Cell culture

Human NSCLC lines (H460, NCI-H520, NCI-H2228, NCI-H3122, PC9, and A549) and a human bronchial epithelial cell line (16HBE) were all purchased from American Type Culture Collection (ATCC, Manassas, VA). The cells were cultured in RPMI-1640 medium (Hyclone, Longan, UT) supplemented with 10% FBS under 37 °C and 5% CO_2_.

### Cell grouping and transduction

Cells were trypsinized, plated in 6-well plates at a density of 1 × 10^5^ cells/well and cultured for 24 h. Upon reaching 75% confluence, cells were subjected to transfection of plasmids or transduction of lentiviral vectors (LV) carrying short hairpin RNA (shRNA) or RNA mimics with Lipofectamine 2000 reagent (Invitrogen, Carlsbad, CA).

A549 cells were treated, respectively, with plasmids containing miR-582-5p mimic, or plasmids carrying shRNA targeting UCHL3/LINC00665 (sh-UCHL3/sh-LINC00665) alone or in combination with LV carrying AhR (LV-AhR), or corresponding negative control (NC), referred to as miR-582-5p mimic, mimic NC, sh-UCHL3, sh-LINC00665, sh-NC, sh-UCHL3 + LV-AhR, sh-UCHL3 + Vector, sh-LINC00665 + LV-AhR, and sh-LINC00665 + Vector groups.

Meanwhile, PC9 cells were treated with miR-582-5p inhibitor, or lentiviral UCHL3/LINC00665 alone or in combination with sh-AhR, or corresponding NC, referred to as miR-582-5p inhibitor, inhibitor NC, LV-UCHL3, LV-LINC00665, Vector, LV-UCHL3 + sh-AhR, LV-UCHL3 + sh-NC, LV-LINC00665 + sh-AhR, and LV-LINC00665 + sh-NC groups. After 8-h transfection, the cells were exposed to irradiation (0, 2, 4, and 6 Gy). The concentration of the used plasmid vectors was 50 ng/mL, and that of shRNA-containing plasmids was completed by Sangon Biotech.

### Western blot analysis

High-efficiency RIPA lysis buffer supplemented with 1% protease inhibitor and 1% phosphorylase inhibitor (Beyotime, Shanghai, China) was adopted for total protein extraction from tissues and cells. Following protein concentration measurement using a BCA kit (Thermo Fisher Scientific), sample proteins were separated by 10% SDS-PAGE, transferred to a PVDF membrane, and then blocked with 5% BSA. Subsequently, the protein-loaded membrane was probed overnight at 4 °C with primary antibodies to UCHL3 (rabbit, 1:2000, ab241490, Abcam), AhR (mouse, 1:500, MA1-513, Thermo Fisher Scientific), PD-L1 (rabbit, 1:1000, ab205921, Abcam) and β-actin (rabbit, 1:5000, ab8227, Abcam; loading control), followed by 1.5-h incubation with HRP-labeled goat anti-rabbit IgG (1:20000, ab205718, Abcam) or HRP-labeled goat anti-mouse IgG (1:500, 31430, Thermo Fisher Scientific) at room temperature. The blots were visualized using developing solution (NCI4106, Pierce, Rockford, IL). Quantitative analysis was then conducted using ImageJ 1.48u software (Bio-Rad, HercμLes, CA).

### Cyclohexylimide (CHX) pulse-chase analysis

CHX (10 μg/mL, SIH-247-1G, Amyjet Scientific, Wuhan, Hubei, China) was added to A549 and PC9 cells to repress protein synthesis, followed by the Western blot analysis to monitor the time required for the degradation of the target protein AhR.

### Co-immunoprecipitation (Co-IP) assay

Co-IP assay was performed to evaluate the interaction between UCHL3 and AhR. HEK293T cells (ATCC) were lysed in IP lysis buffer (P0013). Next, the cell lysate containing 200 μg of protein was then incubated with Dynabeads^®^ protein G magnetic beads at 4 °C for 4 h, with 2 μg of anti-AhR antibody (sc-133088, Santa Cruz Biotechnology, Santa Cruz, CA) or IgG (as the NC) added in. Then, the precipitated protein complexes underwent western blot analysis with the anti-rabbit antibody against UCHL3 (1:2000, ab241490, Abcam).

### Ubiquitination detection

UCHL3-treated A549 and PC9 cells were immunoprecipitated with 2 μg of anti-AhR antibody, followed by the detection of ubiquitination of the AhR protein utilizing the anti-Ub antibody. Subsequent analysis of ImageJ 1.48u software (Bio-Rad) was conducted for quantitation of protein.

### CCK-8 assay

Transduced A549 and PC9 cells were seeded (1 × 10^4^ cells/well) into 96-well plates, and then irradiated at different doses (0, 2, 4, 6 Gy) for 48 h, followed by the detection of cell viability using a CCK-8 kit (K1018, Apexbio, Shanghai, China). Briefly, 10 μL of CCK-8 solution was added to each well for 2-h incubation at 37 °C. A Multiskan FC microplate reader (51119080, Thermo Fisher Scientific) was adopted to examine optical density (OD) at a 450 nm wavelength.

### Colony formation assay

Cells seeded (200 cells/well) in 6-well plates were cultured for 24 h, irradiated (6 Gy) and cultured for another 14 days. After that, the cells were fixed with 4% paraformaldehyde and stained with 0.5% crystal violet. Images were photographed to count the number of colonies formed.

### Transwell invasion assay

An 8-mm pore size Transwell system (Corning, Lowell, MA) with 24-well plates was used for in vitro detection of cell invasion. Specifically, 600 μL of 20% FBS RPMI 1640 medium was added to the lower chamber of the Transwell system with Matrigel-coated basement membrane. The cells, after 48-h transfection, were resuspended in RPMI 1640 medium containing 10% FBS, and 100 μL of the cell suspension (1 × 10^9^ cells/L) to the upper chamber, followed by 24-h incubation at 37 °C and 5% CO_2_. Then, the total invaded cells were subjected to 4% methanol fixing, 0.1% crystal violet staining, and observation under an inverted microscopy.

### Immunofluorescence

Cell sections were blocked with 10% BSA at 25 °C for 1 h and incubated with rabbit anti-γ-H2ax primary antibody (1:400, 9718, Cell Signaling Technology, Boston, MA) at 4 °C for 12–16 h. The sections were incubated with Alexa Fluor 647 donkey anti-rabbit IgG secondary antibody (Thermo Fisher Scientific) at 25 °C for 1 h, followed by staining with DAPI (10 μg/mL, Sigma-Aldrich) at 25 °C. Stained sections were observed by an Olympus BX51 fluorescence microscopy or a laser scanning confocal microscopy.

### Flow cytometric detection of cell cycle and apoptosis

Cell apoptosis was investigated using Annexin V-APC/PI Apoptosis Detection Kit and Cell Cycle Detection Kit (KeyGen Biotech, Nanjing, China). The cells were irradiated with 6 Gy X-rays and incubated with 5 μL Annexin V-APC and 1 μL PI working solution (100 μg/mL) for 15 min at room temperature. Acquired data were analyzed by the FlowJo software.

### Dual-luciferase reporter assay

The miR-582-5p binding sites to UCHL3 were predicted with the TargetScan tool, and the LINC00665 binding sites to miR-582-5p were predicted with DIANA TOOLS. The wild-type (WT) and mutant (Mut) sequences of LINC00665 were constructed, respectively; the WT sequence for the target site in the 3'-UTR region of UCHL3 mRNA, and the corresponding Mut sequence, were constructed, which were then inserted into the pGL-3 luciferase vector (Realgene, Shanghai, China). The luciferase reporter and miR-671-5p mimics or mimics NC were co-transfected into 293 T cells. After 48 h of transfection, luciferase activity was detected using the Luciferase Reporter Assay System (Dual-Luciferase® Reporter Assay System, E1910, Promega, Madison, WI), as normalized to luciferase activity of Renilla luciferase.

### RNA-binding protein immunoprecipitation (RIP) assay

The RIP kit (Millipore) was used to assess the binding of miR-671-5p and LINC00665 to Ago2 protein. An equal volume of RIPA lysis buffer (P0013B, Beyotime) was used to lyse the cells, followed by 10-min centrifugation (14,000 rpm, 4 °C). A part of the cell extract was taken as Input, and the other part was incubated with antibody for co-precipitation assay. For each co-precipitation system, 50 μL of magnetic beads was re-suspended with 100 μL of RIP Wash Buffer and incubated with 5 μg of the corresponding antibody; the magnetic bead-antibody complex was resuspended in 900 μL RIP Wash Buffer and then incubated with 100 μL cell extract overnight at 4 °C. Subsequently, the magnetic bead-conjugated protein was digested with proteinase K, and total RNA was extracted for subsequent PCR analysis. The antibodies used in this assay were as follows: rabbit anti-human Ago2 (ab186733, 1:50, Abcam) and rabbit anti-human IgG (ab109489, 1:100, Abcam) as the NC.

### RNA pull-down assay

Mononuclear cells were transfected with 50 nM biotin-labeled Bio-miR-582-5p-WT and Bio-miR-582-5p-MUT (Genecreate, Wuhan, Hubei, China). After 48 h, the cells were lysed and the lysate was subsequently incubated with M-280 streptavidin magnetic beads (S3762, Sigma-Aldrich) pre-coated with RNase-free BSA and yeast tRNA (TRNABAK-RO, Sigma-Aldrich). After washing with pre-cooled lysis buffer, low-salt buffer, and high-salt buffer, the bound RNA was purified by Trizol reagent, followed by qRT-PCR detection of the enrichment of UCHL3 and LINC00665.

### Animal experiments

A total of 40 6-week-old male C57BL/6 mice (weighing 18—22 g, purchased from Beijing Institute of Pharmacology, Chinese Academy of Medical Sciences, Beijing, China) were housed separately in a specific pathogen-free animal laboratory (60—65% humidity, 22—25 °C) with free access to food and water under a 12-h light/dark cycle. After one week of acclimatization, a xenograft model of NSCLC was established through subcutaneous inoculation of the stably transduced PC9 cells (5 × 10^6^ cells, 100 μL; LV-LINC00665, alone or in combination with sh-AhR) into the back and abdomen of mice (n = 10 in each group).

As described previously, 10 Gy X-rays can effectively kill NSCLC cells in mice [19], so we chose 10 Gy X-rays to irradiate the mouse models. A Varian Clinac 600C X-ray device was used for irradiation at 250 cGy/min (80 cm away from the skin). Before irradiation, the mice were anesthetized with isoflurane inhaled and shielded with a lead cover to ensure that only the xenografted tumor was exposed to radiation. When the tumor volume reached 70 mm^3^, the mice were irradiated weekly with 10 Gy X-ray for a total of three weeks. After 28 days, the animals were euthanized by 1% pentobarbital sodium, and the tumor was isolated and weighed. The tumor volume was calculated based on the following formula: tumor volume = (length × width^2^)/2.

### TUNEL staining

The TUNEL Apoptosis Detection Kit (Millipore, Billerica, MA) was utilized to detect apoptosis in tumor tissues [20]. Sections were treated with 20 μg/mL DNase-free proteinase K (ST532, Beyotime) and 3% hydrogen peroxide solution. Then, the sections were incubated with biotin labeling solution at 37 °C for 60 min. After 30-min incubation with 50 μL streptavidin-HRP working solution, the sections were then treated with DAB for color development and later subjected to microscopic observation to determine TUNEL-positive cells (with brown-yellow nuclei).

### Multiplexed immunohistochemistry (mIHC)

An Opal 7-color Fluorescence Immunohistochemistry (IHC) Kit (PerkinElmer, Waltham, MA) was utilized for the mIHC assay, with the following primary antibodies involved: anti-CD8 (1:800, ab93278, Abcam), PD-1 (1:800, ab137132, Abcam), D240 (1:500, M361929-2, Dako, Glostrup, Denmark) and PD-L1 (5 μg/mL, ab205921, Abcam). Sections were incubated with the HRP Broad Spectrum SuperPicture Polymer Detection Kit (Thermo Fisher Scientific) and diluted with opal fluorescent dyes (Opal520, Opal570, Opal 620 and Opal 690). Afterwards, the sections were microwaved with AR6 buffer, and incubated with DAPI, followed by observation under a Zeiss LSM 880 confocal laser scanning microscopy.

### Enzyme-linked immunosorbent assay (ELISA)

The mouse tumor tissue was homogenized and centrifuged, with the supernatant collected. ELISA kit was used to detect the production of INF-γ (MIF00, BD Bioscience, San Jose, CA) and TNF-α (MTA00B, BD Bioscience) following the manufacturer’s instructions.

### Statistical analysis

Statistical analysis was conducted by SPSS 21.0 software (IBM, Armonk, New York). Measurement data were summarized as mean ± standard deviation. Paired *t* test was conducted for comparisons between tumor tissues and adjacent normal tissues, and other comparisons between two groups were conducted with independent sample *t* test. One-way analysis of variance (ANOVA) with Tukey's post hoc test was conducted for multiple group comparison. Two-way analysis of variance (ANOVA) with Tukey's post hoc test was conducted for data at different time points among multiple groups. Statistical significance was set up at *p* < 0.05.

## Results

### UCHL3 is up-regulated in NSCLC tissues and cells, and this upregulation correlates with poor outcomes in NSCLC patients

**Fig. 1:**
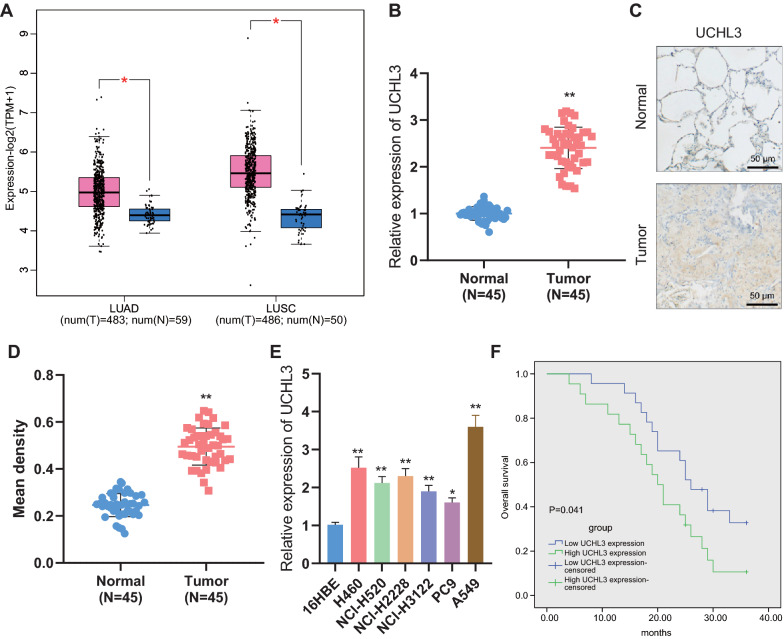
UCHL3 expression was elevated in NSCLC tissues and cells. **A**, The expression of UCHL3 in TCGA-LUAD and TCGA-LUSC datasets from the GEPIA database (T represents tumor tissue, displayed as a red column; N represents normal tissue, displayed as a blue column). **B**, qRT-PCR measurement of UCHL3 expression in tumor tissues and adjacent normal tissues from NSCLC patients (n = 45). **C**, Positive expression of UCHL3 protein in tumor tissues and adjacent normal tissues from NSCLC patients (n = 45) detected by Immunohistochemistry. **D**, Quantitative results of panel C. **E**, qRT-PCR measurement of UCHL3 expression in NSCLC cell lines (H460, NCI-H2228, NCI-H3122, PC9 and A549) and human bronchial epithelial cell line 16HBE. **F**, Correlation between the expression of UCHL3 and the survival rate of NSCLC patients analyzed by the Kaplan–Meier method. * *p* < 0.05, ** *p* < 0.01 versus adjacent normal tissues or 16HBE cells. Each cellular experiment was repeated 3 times

Initial results of the bioinformatics analysis based on GEPIA and TCGA databases revealed that UCHL3 was up-regulated in NSCLC tissues, relative to normal tissues (Fig. 1A).

Further, we validated through qRT-PCR the elevated UCHL3 levels in NSCLC tissues (referred to as the Tumor group) compared with adjacent normal tissues (referred to as the Normal group) (Fig. 1B). Immunohistochemistry results also displayed an increased rate of UCHL3-positive cells in NSCLC tissues (Fig. 1C, D). Moreover, UCHL3 expression was also shown to be up-regulated in NSCLC cell lines (H460, NCI-H2228, NCI-H3122, PC9 and A549), relative to that in human bronchial epithelial 16HBE cells. Among the 5 NSCLC cell lines, A549 cells presented with the highest UCHL3 expression and PC9 cells presented with the lowest UCHL3 expression (Fig. 1E). Herein, A549 and PC9 cells were selected for following investigations. Moreover, the Kaplan–Meier method demonstrated that the abundant expression of UCHL3 indicated a poor survival rate of NSCLC patients (Fig. 1F).

Together, up-regulation of UCHL3 occurred in both NSCLC tissues and NSCLC cells, and that this up-regulation was associated with unfavorable prognosis of NSCLC patients.

### UCHL3 promotes stabilization of AhR protein through deubiquitination, thereby elevating PD-L1 expression

**Fig. 2:**
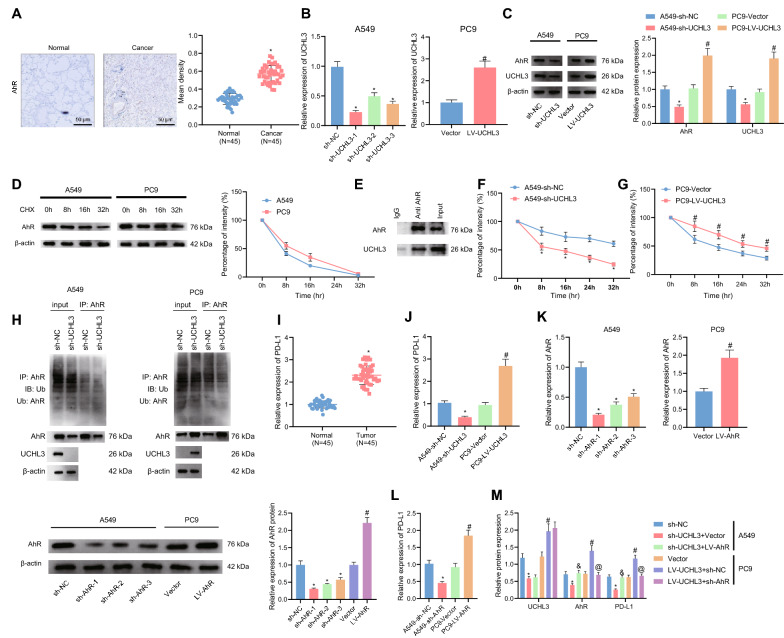
UCHL3 maintained the stability of AhR protein through deubiquitination, thereby promoting PD-L1 expression. **A**, Positive expression of AhR protein in tumor tissues and adjacent normal tissues from NSCLC patients (n = 45) detected by Immunohistochemistry. **B**, Silencing efficiency of sh-UCHL3 in A549 cells and overexpression efficiency of LV-UCHL3 in PC9 cells detected by qRT-PCR. **C**, Protein levels of UCHL3 and AhR in A549 cells treated with sh-UCHL3 or in PC9 treated with LV-UCHL3 detected by Western blot analysis. **D**, Western blot analysis of AhR protein degradation in A549 and PC9 cells following CHX (10 μg/mL) treatment (the left penal) and corresponding statistical analysis of AhR protein stability (the right penal). **E**, Co-IP detection of exogenous UCHL3 and AhR proteins in HEK293T cells. F-G, AhR protein degradation rate in A549 cells in response to sh-UCHL3 (**F**) or in PC9 cells in response to LV-UCHL3 (**G**) detected by CHX pulse-chase analysis. **H**, Detection of AhR protein ubiquitination in A549 cells overexpressing UCHL3 or in PC9 cells silencing UCHL3. **I**, Expression of PD-L1 in tumor tissues and adjacent normal tissues from NSCLC patients (n = 45) detected by qRT-PCR. **J**, Expression of PD-L1 in A549 cells treated with sh-UCHL3 or in PC9 cells treated with LV-UCHL3 detected by qRT-PCR. **K**, Silencing efficiency of sh-AhR in A549 cells and overexpression efficiency of LV-AhR in PC9 cells detected by qRT-PCR (the left two panels) and Western blot analysis (the right two panels). **L**, Expression of PD-L1 in A549 cells treated with sh-AhR or in PC9 cells treated with LV-AhR detected by qRT-PCR. **M**, Protein levels of UCHL3, AhR and PD-L1 in A549 cells treated with sh-UCHL3 or combined with LV-AhR or in PC9 cells treated with LV-UCHL3 or combined with sh-AhR detected by Western blot analysis. * *p* < 0.05 versus the adjacent normal tissues or A549 cells treated with sh-NC; # *p* < 0.05 versus the PC9 cells treated with Vector; & *p* < 0.05 versus the A549 cells treated with sh-UCHL3 + Vector; @ *p* < 0.05 versus the PC9 cells treated with LV-UCHL3 + sh-NC. Each cellular experiment was repeated 3 times

Based on previous documentation on UCHL3 maintaining NSCLC cancer cell stemness by stabilizing AhR protein level via deubiquitination [11], we then determined AhR protein with immunohistochemistry. Increased positive expression AhR protein was witnessed in NSCLC tissues, as compared with adjacent normal tissues (Fig. 2A). qRT-PCR results exhibited that UCHL3 expression was reduced in A549 cells in response to sh-UCHL3 (sh-UCHL3-1, sh-UCHL3-2 and sh-UCHL3-3), among which, sh-UCHL3-1 presented with the optimal silencing efficiency and was thus used for subsequent experiments (Fig. 2B). Besides, overexpression efficiency of UCHL3 in PC9 cells was also validated (Fig. 2B). Silencing UCHL3 led to a reduced level of AhR protein, and UCHL3 overexpression led to an elevated AhR protein level (Fig. 2C).

To determine whether UCHL3 stabilized AhR protein through deubiquitination, we treated the cells with CHX to track the degradation of AhR protein using Western blot analysis. The results displayed that AhR protein was gradually degraded in response to CHX treatment (Fig. 2D). Then, Co-IP results confirmed that AhR interacted with UCHL3 (Fig. 2E). Following CHX treatment, the half-life of AhR protein was found to be shortened in the presence of UCHL3 knockdown, and prolonged in the presence of UCHL3 overexpression (Fig. 2F, G).

Additionally, we directly detect the ubiquitination of endogenous polyubiquitinated AhR protein. The results indicated that UCHL3 knockdown led to augmented ubiquitination of endogenous AhR protein while UCHL3 up-regulation led to the opposite (Fig. 2H). Collectively, our data supported that UCHL3 stabilized AhR protein through promoting deubiquitination.

We then examined the speculation that AhR regulated PD-L1 expression. Elevated expression of PD-L1 was witnessed in NSCLC tissues, and PD-L1 expression was diminished in NSCLC cells with sh-UCHL3 and up-regulated in the presence of UCHL3 overexpression (Fig. 2I, J). Further, knockdown of AhR in A549 cells and overexpressing AhR in PC9 cells were achieved by sh-AhR and LV-AhR, respectively, as validated by qRT-PCR and Western blot analysis. sh-AhR-1 was selected for following investigations for the most notable silencing effects (Fig. 2K). Knockdown of AhR was observed to diminish levels of PD-L1, and AhR overexpression led to an opposite result (Fig. 2L). According to Western blot analysis results, protein levels of UCHL3, AhR and PD-L1 were all reduced in response to sh-UCHL3 treatment, while additional LV-AhR restored the protein levels of AhR and PD-L1 but showed on obvious effects on the UCHL3 protein level; consistently, LV-UCHL3 led to up-regulated protein levels of UCHL3, AhR and PD-L1, among which only the up-regulation of AhR and PD-L1 was reversed in response to additional sh-AhR treatment (Fig. 2M).

In summary, these results unraveled that UCHL3 augmented the stability of AhR protein through deubiquitination, thereby promoting PD-L1 expression.

### UCHL3 potentiates the resistance of NSCLC cells to radiotherapy by stabilizing AhR protein

Following the aforementioned identification of the UCHL3/AhR/PD-L1 cascade, we then managed to investigate the role of the regulatory cascade in the radiosensitivity of NSCLC cells. We exposed A549 and PC9 cells to different doses of radiation following the manipulation of UCHL3 expression.

**Fig. 3:**
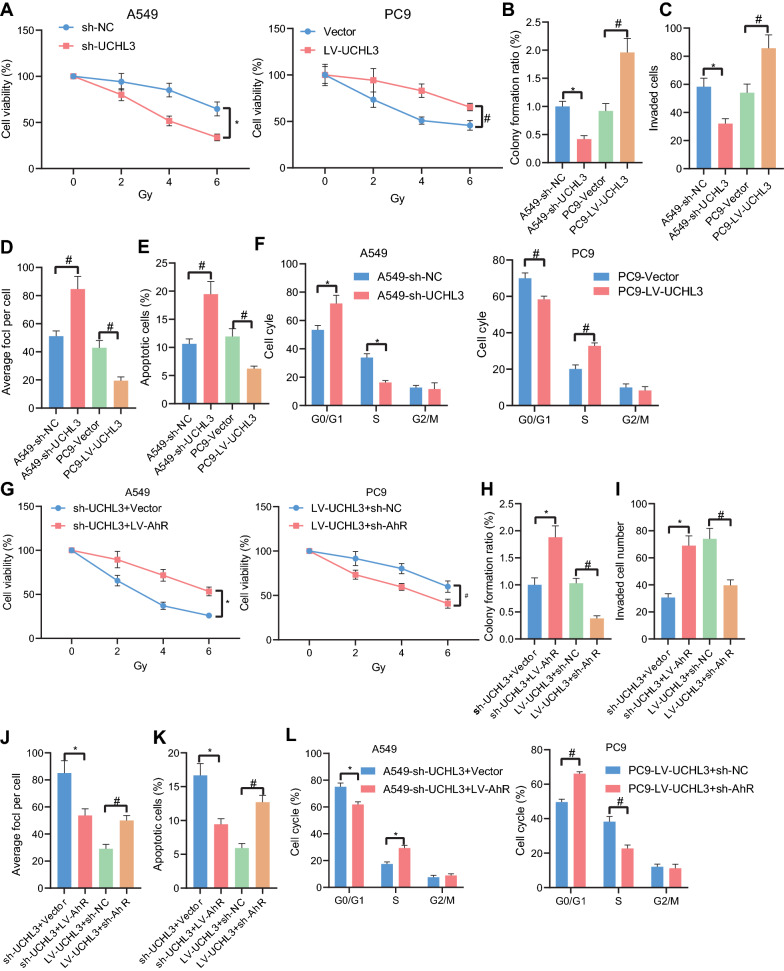
UCHL3 reduced the radiosensitivity of NSCLC cells by stabilizing AhR protein. **A**, Cell viability in A549 cells silencing UCHL3 and in PC9 cells overexpressing UCHL3 measured by CCK-8 assay. **B**, Colony formation potential of A549 cells treated with sh-UCHL3 or in PC9 cells treated with LV-UCHL3 measured by colony formation assay. **C**, Cell invasion in A549 cells treated with sh-UCHL3 or in PC9 cells treated with LV-UCHL3 measured by Transwell assay. D, Detection of γH2ax fluorescence in A549 cells treated with sh-UCHL3 or in PC9 cells treated with LV-UCHL3. E, Flow cytometry analysis of cell apoptosis in A549 cells treated with sh-UCHL3 or in PC9 cells treated with LV-UCHL3. **F**, Flow cytometry analysis of cell cycle distribution in A549 cells treated with sh-UCHL3 or in PC9 cells treated with LV-UCHL3. **G**, Cell viability in A549 cells treated with sh-UCHL3 or combined with LV-AhR or in PC9 cells treated with LV-UCHL3 or combined with sh-AhR measured by CCK-8 assay. **H**, Colony formation potential of A549 cells treated with sh-UCHL3 or combined with LV-AhR or in PC9 cells treated with LV-UCHL3 or combined with sh-AhR measured by colony formation assay. **I**, Cell invasion in A549 cells treated with sh-UCHL3 or combined with LV-AhR or in PC9 cells treated with LV-UCHL3 or combined with sh-AhR measured by Transwell assay. **J**, Detection of γH2ax fluorescence in irradiation-treated A549 cells treated with sh-UCHL3 or combined with LV-AhR or in PC9 cells treated with LV-UCHL3 or combined with sh-AhR. **K**, Flow cytometry analysis of cell apoptosis in A549 cells treated with sh-UCHL3 or combined with LV-AhR or in PC9 cells treated with LV-UCHL3 or combined with sh-AhR. **L**, Flow cytometry analysis of cell cycle distribution in A549 cells treated with sh-UCHL3 or combined with LV-AhR or in PC9 cells treated with LV-UCHL3 or combined with sh-AhR. *, ^#^ *p* < 0.05. Each cellular experiment was repeated 3 times

CCK-8 assay results showed attenuated cell viability in response to silencing UCHL3 as well as augmented cell viability in response to overexpression of UCHL3 (Fig. 3A). Under irradiation (6 Gy), the colony formation and invasive potential of the cells was weakened in the presence of UCHL3 knockdown, and enhanced by UCHL3 overexpression (Fig. 3B, C). In γH2ax immunofluorescence, silencing UCHL3 resulted in increased fluorescence intensity of γH2ax, and UCHL3 overexpression led to reduced intensity (Fig. 3D). Results of flow cytometry then showed that UCHL3 knockdown augmented cell apoptosis, along with increased cells arrested in the G0/G1 phase, decreased cells arrested in the S phase, and repressed cell division, while the restoration of UCHL3 caused opposite results (Fig. 3E, F).

According to results of further gain- and loss- of function assays under irradiation, AhR overexpression reversed the sh-UCHL3-induced suppression on cell viability, and AhR knockdown reversed the promoting effects of LV-UCHL3 on cell viability (Fig. 3G). Similarly, the attenuated cell colony formation and invasion, increased γH2ax immunofluorescence and enhanced cell apoptosis, in the presence UCHL3 knockdown alone, were negated by its combination with AhR restoration, and the effects of UCHL3 overexpression alone were also reversed by additional silencing of AhR (Fig. 3H–L).

Together, these results indicated that UCHL3 enhanced the radioresistance of NSCLC cells by stabilizing AhR protein.

### LINC00665 sponges miR-582-5p to up-regulate UCHL3 expression

**Fig. 4:**
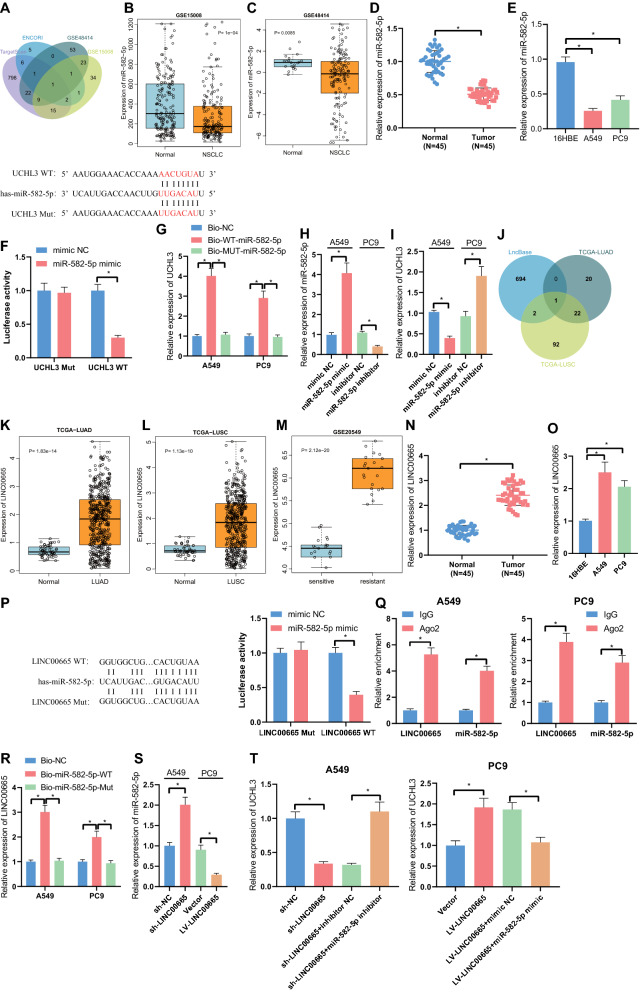
LINC00665 functioned as a sponge of miR-582-5p and thus up-regulated the expression of UCHL3. **A**, Venn diagram of the UCHL3 binding miRNAs predicted by TargetScan and ENCORI databases, respectively, and the poorly expressed miRNAs in lung cancer samples in the GSE15008 and GSE48414 datasets. **B**, The expression of miR-582-5p in NSCLC samples (n = 187) and normal samples (n = 188) in the GSE15008 dataset. **C**, The expression of miR-582-5p in NSCLC samples (n = 154) and normal samples (n = 20) in the GSE48414 dataset. **D**, qRT-PCR measurement of miR-582-5p expression in tumor tissues and adjacent normal tissues from NSCLC patients (n = 45). **E**, qRT-PCR measurement of miR-582-5p expression in NSCLC cells. **F**, Binding of miR-582-5p to UCHL3 verified by dual luciferase reporter assay. **G**, Binding of miR-582-5p to UCHL3 verified by RNA pull-down assay. **H**, qRT-PCR measurement of the transfection efficiency of miR-582-5p mimic and miR-582-5p inhibitor in NSCLC cells; **I**, qRT-PCR measurement of UCHL3 expression in NSCLC cells in response to miR-582-5p mimic or miR-582-5p inhibitor. **J**, Venn diagram of the miR-582-5p binding lncRNAs predicted by the LncBase database and the highly expressed lncRNAs in lung cancer tissues in TCGA-LUAD and in TCGA-LUSC datasets. **K**, The expression of LINC00665 in LUAD samples (n = 526) and normal samples (n = 59) in the TCGA-LUAD dataset. **L**, The expression of LINC00665 in LUSC samples (n = 501) and normal samples (n = 49) in the TCGA-LUSC dataset. **M**, The expression of LINC00665 in radiotherapy-resistant H1299 lung cancer cell lines (n = 21) as well as radiotherapy-sensitive H460 lung cancer cell lines (n = 21) in the GSE20549 dataset. **N**, qRT-PCR measurement of LINC0065 expression in tumor tissues and adjacent normal tissues from NSCLC patients (n = 45). **O**, qRT-PCR measurement of LINC00665 expression in NSCLC cells. **P**, Binding of LINC00665 to miR-582-5p verified by dual luciferase reporter assay. **Q**, Binding of LINC00665 and miR-582-5p to Ago2 in A549 and PC9 cells verified by RIP assay. **R**, Binding of LINC00665 to miR-582-5p in A549 and PC9 cells determined by RNA pull-down assay. **S**, qRT-PCR measurement of miR-582-5p expression A549 cells treated with sh-LINC00665 or in PC9 cells treated with LV-LINC00665. **T**, qRT-PCR measurement of UCHL3 expression in A549 cells treated with sh-LINC00665 or combined with miR-582-5p inhibitor or in PC9 cells treated with LV-LINC00665 or combined with miR-582-5p mimic. * *p* < 0.05. Each cellular experiment was repeated 3 times

Further to explore the upstream mechanism of UCHL3 regulating radiotherapy sensitivity in NSCLC, we conducted bioinformatics analysis to predict miRNAs targeting UCHL3 and identified miR-582-5p that was poorly expressed in NSCLC as the candidate miRNA (Fig. 4A–C). qRT-PCR results identified the down-regulated levels of miR-582-5p in NSCLC tissues relative to normal tissues, and also in NSCLC cells versus 16HBE cells (Fig. 4D, E).

The binding site of miR-582-5p and UCHL3 was predicted with the TargetScan database. Further, the binding of miR-582-5p to UCHL3 was validated by the dual luciferase reporter assay (Fig. 4F) and the RNA pull-down assay (Fig. 4G). Lower luciferase activity was witnessed in response to co-transfection of miR-582-5p mimic and UCHL3 WT as compared to the mimic NC, but no notable difference was observed in response to UCHL3 MUT (Fig. 4F). UCHL3 binding was notably increased in the Bio-WT-miR-582-5p group, relative to Bio-NC (Fig. 4G). Then, miR-582-5p expression was successfully elevated or reduced in response to mimic or inhibitor, as shown in qRT-PCR results (Fig. 4H). The up-regulation of miR-582-5p was shown to diminish UCHL3 levels, and miR-582-5p inhibition augmented UCHL3 expression (Fig. 4I). Thus, miR-582-5p targeted and negatively regulated UCHL3.

Continuing to uncover the upstream gene of miR-582-5p, we used the LncBase database to predict lncRNAs binding to miR-582-5p, of which only LINC00665 was up-regulated in TCGA-LUAD and TCGA-LUSC datasets (Fig. 4J–L). In addition, microarray profiling suggested that LINC00665 was differentially expressed in radioresistant samples relative to radiosensitive samples in lung cancer (Fig. 4M). Therefore, we speculated that LINC00665 may act as a sponge of miR-582-5p to regulate UCHL3 expression.

To examine the speculation, we first confirmed the up-regulated levels of LINC00665 in the NSCLC tissues and cells (Fig. 4N, O). The binding site of LINC00665 and miR-582-5p was predicted using the DIANA TOOLS website. Results of dual luciferase experiments showed that miR-582-5p overexpression reduced the luciferase activity of the WT-LINC00665 group but did not affect that of the MUT-LINC00665 group (Fig. 4P). RIP results showed that Ago2 could enrich LINC00665 and miR-582-5p simultaneously (Fig. 4Q). The results of RNA pull-down assay displayed the increased levels of LINC00665 in A549 and PC9 cells transfected with Bio-miR-582-5p-WT (Fig. 4R). Moreover, knockdown of LINC00665 in A549 cells resulted in elevated miR-582-5p levels, and overexpressing LINC00665 in PC9 cells led to reduced miR-582-5p expression (Fig. 4S). Thus, LINC00665 bound to miR-582-5p and functioned as a miR-582-5p sponge.

Furthermore, sh-LINC00665 treatment alone in A549 cells diminished UCHL3 levels, while its combination with miR-582-5p inhibitor in A549 cells restored UCHL3 expression. The promoting effect of LV-LINC00665 treatment on UCHL3 expression in the PC9 cells was reversed in response to additional treatment by miR-582-5p mimic (Fig. 4T).

Collectively, the aforementioned results revealed that LINC00665 functioned as a sponge of miR-582-5p and thus up-regulated UCHL3.

### Silencing LINC00665 or overexpressing miR-582-5p enhances the sensitivity of NSCLC cells to radiotherapy

**Fig. 5:**
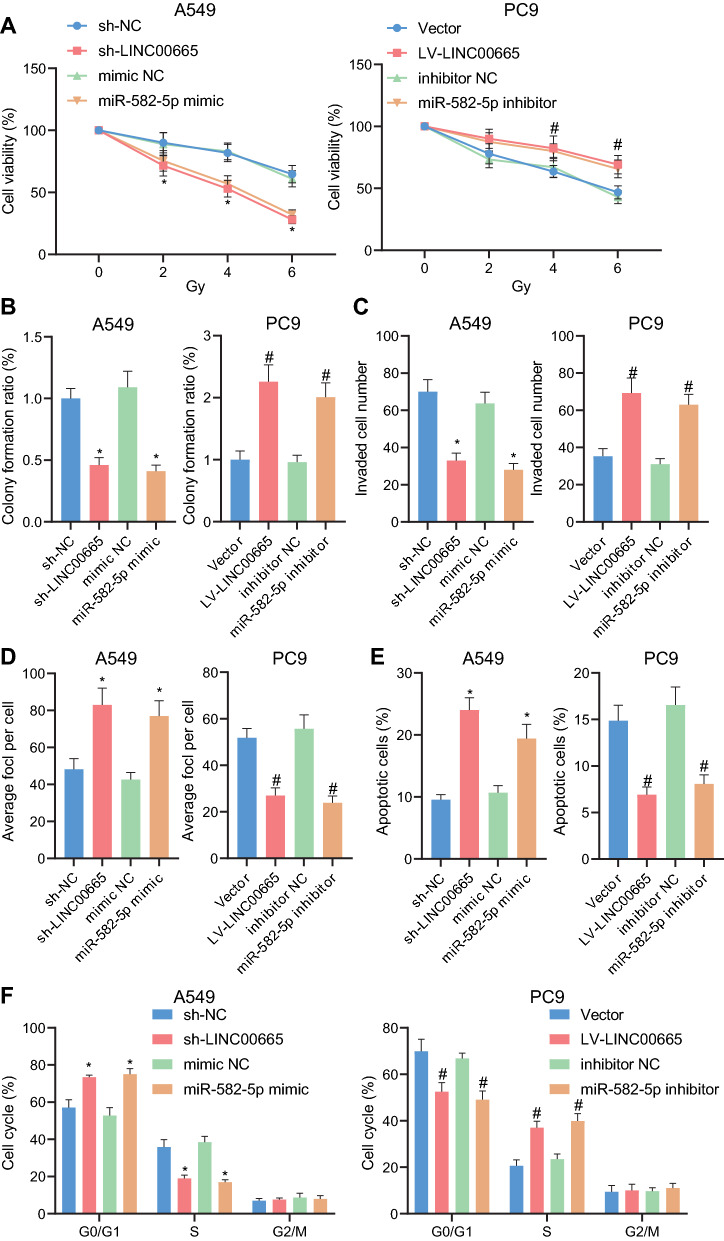
Silencing LINC00665 or overexpressing miR-582-5p enhanced the sensitivity of NSCLC cells to radiotherapy. **A**, Cell viability in A549 cells treated with sh-LINC00665 or miR-582-5p mimic or in PC9 cells treated with LV-LINC00665 or miR-582-5p inhibitor determined by CCK-8 assay. **B**, Colony formation potential of A549 treated with sh-LINC00665 or miR-582-5p mimic or of PC9 cells treated with LV-LINC00665 or miR-582-5p inhibitor determined by colony formation assay. **C**, Cell invasion in A549 cells treated with sh-LINC00665 or miR-582-5p mimic or in PC9 cells treated with LV-LINC00665 or miR-582-5p inhibitor determined by Transwell assay. **D**, Detection of γH2ax fluorescence in A549 cells treated with sh-LINC00665 or miR-582-5p mimic or in PC9 cells treated with LV-LINC00665 or miR-582-5p inhibitor. **E**, Flow cytometry analysis of cell apoptosis in A549 cells treated with sh-LINC00665 or miR-582-5p mimic or in PC9 cells treated with LV-LINC00665 or miR-582-5p inhibitor. **F**, Flow cytometry analysis of cell cycle distribution in A549 cells treated with sh-LINC00665 or miR-582-5p mimic or in PC9 cells treated with LV-LINC00665 or miR-582-5p inhibitor. * *p* < 0.05 versus the A549 cells treated with sh-NC or mimic NC; # *p* < 0.05 versus the PC9 cells treated with Vector or inhibitor NC. Each cellular experiment was repeated 3 times

Following the finding of the LINC00665/miR-582-5p axis, we then looked into its involvement in the radiosensitivity of NSCLC cells. As shown by the results of CCK-8 assay, either silencing LINC00665 or overexpressing miR-582-5p resulted in decreased cell viability while ectopically expressed LINC00665 or depleted miR-582-5p increased the cell viability (Fig. 5A).

According to further functional assays under 6 Gy irradiation, either LINC00665 silencing or miR-582-5p overexpression led to attenuated cell colony formation (Fig. 5B), reduced invasive ability of the cells (Fig. 5C), increased percentage of γH2ax-positive cells (Fig. 5D), and augmented cell apoptosis, alone with augmented G0/G1 cell cycle arrest and less cells in S phase (Fig. 5E, F). Up-regulation of LINC00665 or inhibition of miR-582-5p led to opposite results (Fig. 5B–F).

Together, our data demonstrated that silencing LINC00665 or overexpressing miR-582-5p enhanced the sensitivity of NSCLC cells to radiotherapy.

### LINC00665/miR-582-5p/UCHL3 axis regulates the radiosensitivity via mediating AhR protein stabilization in NSCLC cells

**Fig. 6:**
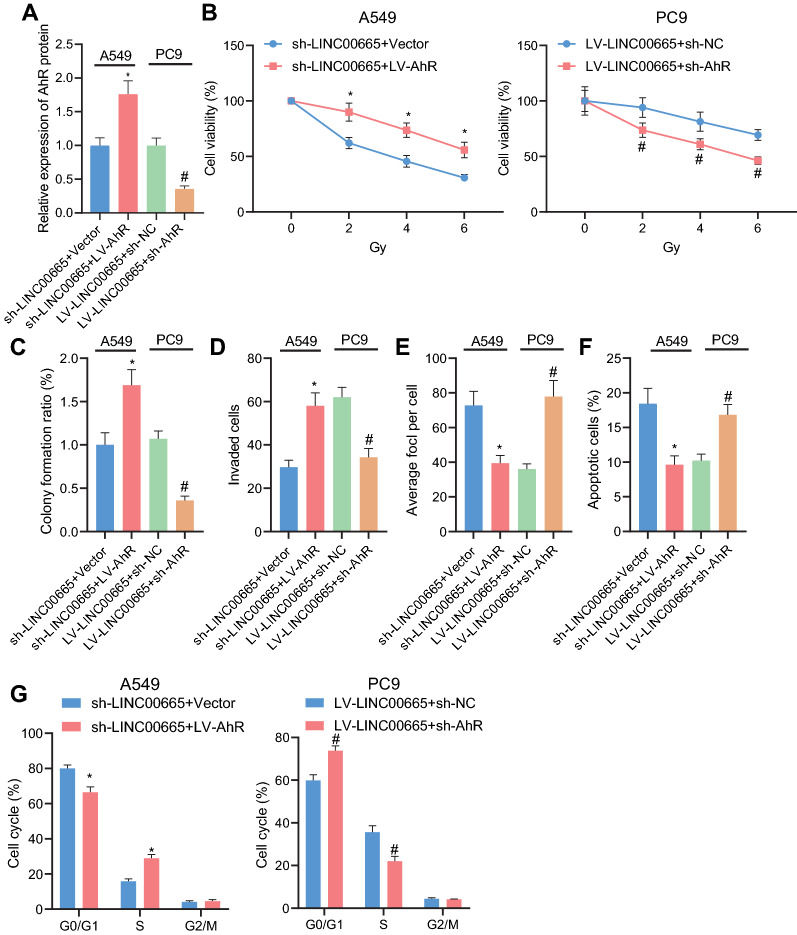
The LINC00665/miR-582-5p/UCHL3 axis affected the radiosensitivity of NSCLC cells by modulating the stability of AhR protein. **A**, Western blot analysis of AhR protein level in A549 cells treated with sh-LINC00665 or combined with LV-AhR or in PC9 cells treated with LV-LINC00665 or combined with sh-AhR. **B**, Cell viability in A549 cells treated with sh-LINC00665 or combined with LV-AhR or in PC9 cells treated with LV-LINC00665 or combined with sh-AhR determined by CCK-8 assay. **C**, Colony formation potential of A549 cells treated with sh-LINC00665 or combined with LV-AhR or in PC9 cells treated with LV-LINC00665 or combined with sh-AhR determined by colony formation assay. **D**, Cell invasion in A549 cells treated with sh-LINC00665 or combined with LV-AhR or in PC9 cells treated with LV-LINC00665 or combined with sh-AhR determined by Transwell assay. **E**, Detection of γH2ax fluorescence in irradiation-treated A549 cells treated with sh-LINC00665 or combined with LV-AhR or in PC9 cells treated with LV-LINC00665 or combined with sh-AhR. **F**, Flow cytometry analysis of cell apoptosis in A549 cells treated with sh-LINC00665 or combined with LV-AhR or in PC9 cells treated with LV-LINC00665 or combined with sh-AhR. **G**, Flow cytometry analysis of cell cycle distribution in A549 cells treated with sh-LINC00665 or combined with LV-AhR or in PC9 cells treated with LV-LINC00665 or combined with sh-AhR. * *p* < 0.05 versus the A549 cells treated with sh-LINC00665 + Vector; # *p* < 0.05 versus the PC9 cells treated with LV-LINC00665 + sh-NC. Each cellular experiment was repeated 3 times

Subsequently, we performed gain- and loss- of function assays to verify that the LINC00665/miR-582-5p/UCHL3 axis affected the radiosensitivity of NSCLC by mediating the stability of the AhR protein. As shown in Western blot analysis results, cells treated with the combination of sh-LINC00665 and LV-AhR presented with elevated AhR protein level relative to sh-LINC00665-treated cells; the combination of LV-LINC00665 and sh-AhR led to decreased AhR protein level as compared with LV-LINC00665 alone (Fig. 6A). Cell viability of sh-LINC00665-treated cells was enhanced by additional LV-AhR treatment, and the viability of LV-LINC00665-treated cells was attenuated in response to additional AhR knockdown (Fig. 6B).

Under 6 Gy irradiation, as compared with silencing LINC00665 alone, its combination with AhR overexpression led to potentiated cell colony formation and invasion, fewer γH2ax-positive cells, and suppressed cell apoptosis accompanied by reduced G0/G1 cell cycle arrest and increased S phase cell-cycle arrest; the combination of LINC00665 overexpression and AhR silencing led to the opposite results (Fig. 6C–G).

Taken together, LINC00665/miR-582-5p/UCHL3 axis affected the radiosensitivity of NSCLC cells by modulating the stability of AhR protein.

### LINC00665/miR-582-5p/UCHL3 axis reduces radiosensitivity and promotes immune escape of NSCLC cells in vivo in an AhR protein stabilization-dependent manner

**Fig. 7:**
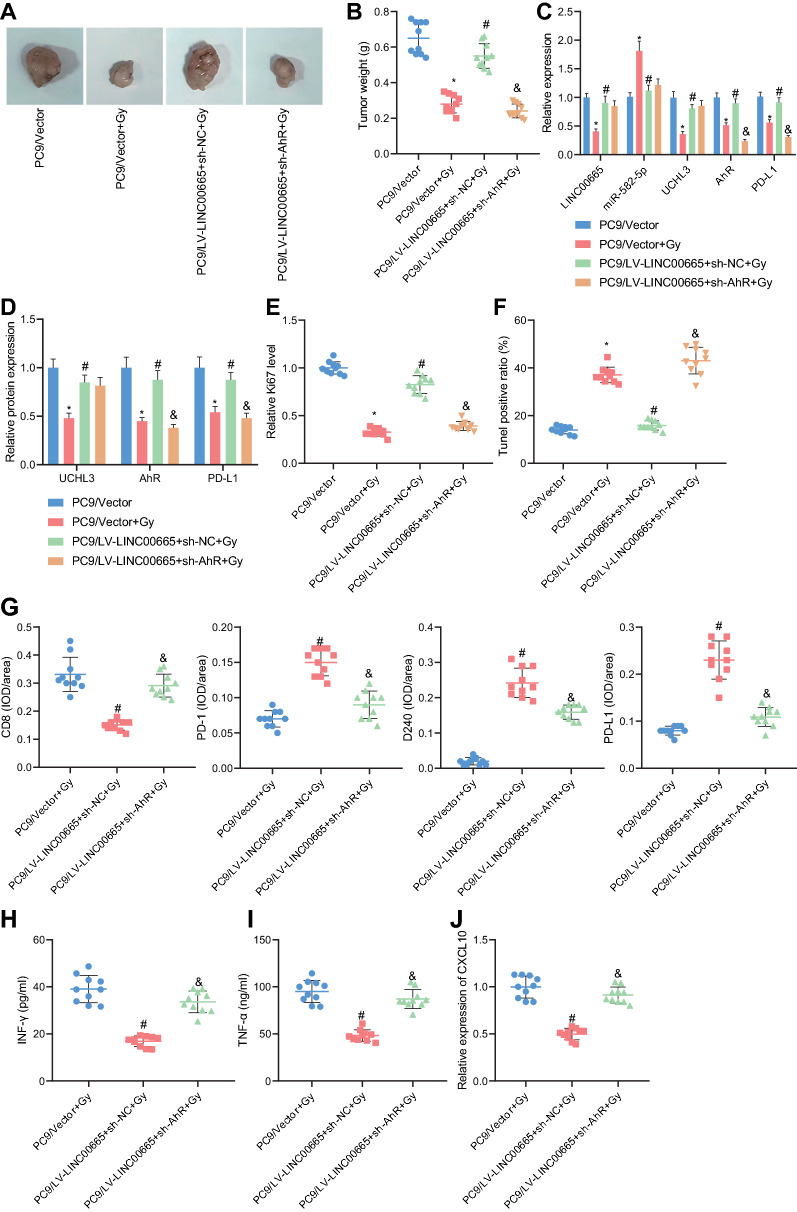
LINC00665 augmented the immune escape of NSCLC cells through the miR-582-5p/UCHL3 regulatory axis by mediating the stability of AhR protein. **A**, Representative images of xenografts in tumor-bearing mice under irradiation treatment and injected with PC9 cells overexpressing LINC00665 alone or in combination with silencing AhR. **B**, Tumor weight of the xenografted tumor in tumor-bearing mice under irradiation treatment and injected with PC9 cells overexpressing LINC00665 alone or in combination with silencing AhR. **C**, qRT-PCR determination of miRNA levels of LINC00665, miR-582-5p, UCHL3, AhR, and PD-L1 in tumor tissues from tumor-bearing mice under irradiation treatment and injected with PC9 cells overexpressing LINC00665 alone or in combination with silencing AhR. **D**, Western blot analysis of protein levels of UCHL3, AhR, and PD-L1 in tumor tissues from tumor-bearing mice under irradiation treatment and injected with PC9 cells overexpressing LINC00665 alone or in combination with silencing AhR. **E**, Detection of ki67-positive cells was to assess tumor cell proliferation in tumor tissues from tumor-bearing mice under irradiation treatment and injected with PC9 cells overexpressing LINC00665 alone or in combination with silencing AhR. **F**, Cell apoptosis in tumor tissues from tumor-bearing mice under irradiation treatment and injected with PC9 cells overexpressing LINC00665 alone or in combination with silencing AhR determined by TUNEL staining. **G**, Representative immunofluorescence images of CD8, PD-1, D240 and PD-L1 in tumor tissue sections (the left panels) and corresponding statistical analysis (the right panel). **H**, **I**, ELISA detection of INF-γ (H) and TNF-α (I) in tumor tissues from tumor-bearing mice under irradiation treatment and injected with PC9 cells overexpressing LINC00665 alone or in combination with silencing AhR. **J**, qRT-PCR measurement of CXCL10 expression in tumor tissues from tumor-bearing mice under irradiation treatment and injected with PC9 cells overexpressing LINC00665 alone or in combination with silencing AhR. n = 10. * *p* < 0.05 versus the mice treated with PC9/Vector; # *p* < 0.05 versus the mice treated with PC9/Vector + Gy; & *p* < 0.05 versus the mice treated with PC9/LV-LINC00665 + sh-NC + Gy

After the in vitro findings, we moved to in vivo substantiation of the mechanism by which the LINC00665/miR-582-5p/UCHL3 axis mediated the stability of AhR protein to affect the radiosensitivity and immune escape in NSCLC. We established a mouse xenograft model of NSCLC, wherein the injection of LINC00665 overexpressing PC9 cells was found to promote the tumor weight in mice under irradiation, and such promoting effects could be reversed by AhR knockdown (Fig. 7A, B).

Furthermore, in the presence of irradiation, the levels of LINC00665, UCHL3, AhR and PD-L1 were up-regulated and miR-582-5p expression was down-regulated in the tumor tissues of mice injected with LINC00665 overexpressing cells; and additional AhR knockdown LINC00665 negated the increase in AhR and PD-L1 expression but showed no effects on miR-582-5p and UCHL3 expression (Fig. 7C).

Meanwhile, Western blot analysis unraveled that LINC00665 overexpression alone led to up-regulated protein levels of UCHL3, AhR and PD-L1 in tumor tissues from irradiation-treated tumor-bearing mice, and its combination with AhR silencing led to relatively decreased protein levels of AhR and PD-L1 and almost unchanged UCHL3 expression (Fig. 7D).

Moreover, tumor tissues from mice treated with PC9/LV-LINC00665 and irradiation presented with an increased rate of ki67-positive cells and a decreased rate of TUNEL-positive cells, which could be reversed by additional AhR knockdown (Fig. 7E, F). Therefore, LINC00665 can stabilize AhR protein through the LINC00665/miR-582-5p/UCHL3 regulatory axis, thus promoting the in vivo tumor formation of NSCLC cells and reducing the radiosensitivity.

Next, we then conducted immunofluorescence to detect CD8 + cell infiltration in the tumor. Tumor tissues of irradiation-treated mice injected with LINC00665 overexpressing cells exhibited fewer CD8 + T cells and elevated levels of PD-1, PD-L1 and lymphatic endothelial marker D240; and the aforementioned tendencies were abrogated in response to AhR knockdown (Fig. 7G). ELISA detection of inflammation-related factors (INF-γ and TNF-α) further showed that tumor tissues from mice treated with LV-LINC00665 and irradiation presented with decreased levels of INF-γ and TNF-α, which could be reversed additional sh-AhR treatment (Fig. 7H, I). Meanwhile, LINC00665 overexpression alone diminished IFN-γ-Inducible Protein-10 (CXCL10) levels, and its combination with the knockdown of AhR reversed the reduction of CXCL10 expression (Fig. 7J).

Collectively, our in vivo data suggested that LINC00665 augmented the immune escape of NSCLC cells through promoting UCHL3-mediated stabilization of AhR protein via the miR-582-5p/UCHL3 regulatory axis.

## Discussion

**Fig. 8:**
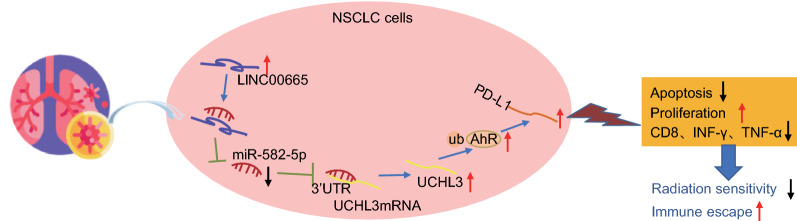
Graphical summary of the regulatory mechanism of LINC00665 in NSCLC radiosensitivity. LINC00665 acted as a sponge of miR-582-5p to inhibit miR-582-5p expression, up-regulated the expression of UCHL3, and enhanced the stability of AhR protein, thereby promoting the immune escape and reducing the radiosensitivity in NSCLC cells

With the aim to boost the efficacy of radiotherapy in NSCLC, this study elucidated a novel mechanism by which the LINC00665 augmented the stabilization of AhR protein through miR-582-5p-mediated regulation of the deubiquitylase UCHL3, thereby facilitating the immune escape of NSCLC cells and reducing radiosensitivity (Fig. 8).

Our initial results identified the up-regulated UCHL3 levels in both NSCLC tissues and cells, and that this upregulation was responsible for the poor prognosis of NSCLC patients. This finding corroborates emerging data supporting the oncogenic role of UCHL3 in NSCLC that could be attributed to its promoting effects on the malignant behaviors on NSCLC cells [21, 22]. Molecular targets hold promising potential for diagnostic and prognostic significance in cancers. For instance, Metadherin (AEG-1/MTDH/LYRIC) has been closely related to the poor prognosis of colorectal cancer, and can be used as a marker for the clinical diagnosis and a potential target for the treatment of colorectal cancer [23, 24]. Serum NRP-1 and ANG-2 levels have been used as markers of hepatocellular carcinoma as their high levels are associated with advanced tumor characteristics [25]. Furthermore, our data unraveled that UCHL3 augmented the stability of AhR protein through deubiquitination, thereby promoting PD-L1 expression. As a deubiquitination, UCHL3 has been suggested to maintain cancer stem-like properties and accelerate tumor cell growth by stabilizing AhR protein [11]. Intriguingly, activation of the AhR signaling pathway has previously been correlated with the radioresistance, and AhR knockout suppresses the malignant phenotypes of cancer cells [26, 27]. Also, in addition to serving as a well-recognized predictive biomarker to guide anti-PD-1/PD-L1 therapy, PD-L1 links to the poorer survival outcomes in metastatic NSCLC [28]. In agreement with these studies, our data illuminated that UCHL3 reduced the radiosensitivity of NSCLC cells by stabilizing AhR protein. Liu *et. al.* also argued that depleted UCHL3 potentiated radiosensitivity of NSCLC cancer cells through potentiating radiation-induced DNA damage [29].

Mechanistic investigations further manifested that LINC00665 functioned as a sponge of miR-582-5p and thus up-regulated UCHL3 levels. LINC00665 is an intensively investigated oncogenic lncRNA in a wide spectrum of cancers, and the tumor-promoting role was generally dependent on its function as a sponge for miRNAs [30–32]. miR-150 serves a potential predictive biomarker for anti-cancer therapy response in chronic myeloid leukemia [33]. In terms of NSCLC, the up-regulated LINC00665 level has been involved in the resistance of NSCLC cells to Gefitinib and Cisplatin [34, 35], and downregulation of LINC00665 attenuates NSCLC cell viability and invasion [17]. Furthermore, we performed a series of functional experiments and demonstrated that silencing LINC00665 or overexpressing miR-582-5p enhanced the sensitivity of NSCLC cells to radiotherapy, and that the LINC00665/miR-582-5p/UCHL3 axis affected the radiosensitivity of NSCLC cells by modulating the stability of AhR protein. In relation to our findings, miR-582-5p has recently been established as a tumor suppressor in NSCLC [18, 36]. Dittmann et al. has uncovered that AhR knockdown leads to obvious radio-sensitization of both human lung carcinoma epithelial (A549) cells and human keratinocyte (HaCaT) cells [37]. Following in vitro investigations, our in vivo data substantiated that LINC00665 augmented the immune escape of NSCLC cells through stabilizing AhR protein via the miR-582-5p/UCHL3 regulatory axis.

## Conclusions

Taken together, experimental data from the present study delineated that LINC00665 acted as a miR-582-5p sponge to up-regulate UCHL3 levels, and enhanced the stability of AhR protein, thereby reducing the radiosensitivity and promoting the immune escape in NSCLC cells. In addition, our data directly confirmed that silencing LINC00665 or overexpressing miR-582-5p enhanced the sensitivity of NSCLC cells to radiotherapy. Herein, this study not only deepened our understanding of the mechanisms underlying the radioresistance of NSCLC cells but also provided novel potential therapeutic targets for the improvement of the efficacy of radiotherapy in NSCLC. Still, further studies with larger sample sizes are needed to confirm the obtained findings of the current study.

## Supplementary Information

**Additional file 1: Table S1.** Primer sequences for qRT-PCR.

## Data Availability

The data and materials of the study can be obtained from the corresponding author upon request.

## Abbreviations

NSCLC: Non-small cell lung cancer
UCHL3: Ubiquitin C-terminal hydrolase L3
miRNAs: MicroRNAs
AhR: Aryl hydrocarbon receptor
DUBs: Deubiquitinating enzymes
UCH: Ubiquitin C-terminal hydrolase
PD-L1: Programmed cell death-ligand 1
lncRNAs: Long non-coding RNAs
GEO: Gene expression omnibus
TCGA: The cancer genome atlas
LUAD: Lung adenocarcinoma
LUSC: Lung squamous cell carcinoma
AJCC: American joint committee on cancer
shRNA: Short hairpin RNA
NC: Negative control
qRT-PCR: Quantitative reverse transcription polymerase chain reaction
cDNA: Complementary DNA
GAPDH: Glyceraldehyde-3-phosphate dehydrogenase
SDS-PAGE: Sodium dodecyl sulfate polyacrylamide gel electrophoresis
HRP: Horseradish peroxidase
RPMI: Roswell park memorial institute
EDTA: Ethylenediamine tetraacetic acid
FBS: Fetal bovine serum
RIPA: Radioimmunoprecipitation assay
BSA: Bovine serum albumin
MUT: Mutant
WT: Wild-type
DAB: 3, 3′-Diaminobenzidine
CHX: Cyclohexylimide
Co-IP: Co-immunoprecipitation
CCK-8: Cell counting kit-8
RIP: RNA-binding protein immunoprecipitation
mIHC: Multiplexed immunohistochemistry
ELISA: Enzyme-linked immunosorbent assay
ANOVA: Analysis of variance
