# Supplementary material for: LINC00665 knockdown confers sensitivity in irradiated non-small cell lung cancer cells through the miR-582-5p/UCHL3/AhR axis
Source: J Transl Med. 2022 Aug 2;20:350. doi: 10.1186/s12967-022-03516-2 (PMC9344728; doi:10.1186/s12967-022-03516-2)
Supplement: Supplementary file 1 — Additional file 1: Table S1. Primer sequences for qRT-PCR. [file 12967_2022_3516_MOESM1_ESM.docx]

**Additional file Table S1 Primer sequences for qRT-PCR.**

| Genes | Primer sequences |
| --- | --- |
| UCHL3 (human) | Forward: 5ʹ-AGAACGAGCCAGATACCTGGA-3′ |
|  | Reverse: 5ʹ-GCTTCCGCCCATCTAATTCAT-3′ |
| AhR (human) | Forward: 5ʹ-ACATCACCTACGCCAGTCG-3′ |
|  | Reverse: 5ʹ-CGCTTGGAAGGATTTGACTTGA-3′ |
| PD-L1 (human) | Forward: 5ʹ-TGGCATTTGCTGAACGCATTT-3′ |
|  | Reverse: 5ʹ-TGCAGCCAGGTCTAATTGTTTT-3′ |
| CXCL10 (human) | Forward: 5ʹ-GTGGCATTCAAGGAGTACCTC-3′ |
|  | Reverse: 5ʹ-TGATGGCCTTCGATTCTGGATT-3′ |
| miR-582-5p (human) | Forward: 5′-TTACAGTTGTTCAACCAGTTACT-3′ |
|  | Reverse: Universal reverse primer of the kit |
| LINC00665 (human) | Forward: 5′-GGTGCAAAGTGGGAAGTGTG-3′ |
|  | Reverse: 5′-CGGTGGACGGATGAGAAACG-3′ |
| GAPDH (human) | Forward: 5′-TCTAGAAAAACCTGCCAAATATG-3′ |
|  | Reverse: 5′-TGGTGCTCAGTGTAGCCCAGGA-3′ |
| U6 (human) | Forward: 5′-CGCTTCGGCAGCACATATACTA-3′ |
|  | Reverse: Universal reverse primer of the kit |

Note: qRT-PCR, quantitative reverse transcription-polymerase chain reaction; UCHL3, Ubiquitin C-terminal hydrolase L3; AhR, Aryl hydrocarbon receptor; PD-L1, programmed cell death receptor 1; miR-582-5p, microRNA-582-5p; GAPDH, glyceraldehyde-3-phosphate dehydrogenase.
